# In Vitro, In Silico, and In Vivo Assessments of Pharmacokinetic Properties of ZM241385

**DOI:** 10.3390/molecules25051106

**Published:** 2020-03-02

**Authors:** Jin-Ju Byeon, Min-Ho Park, Seok-Ho Shin, Yuri Park, Byeong ill Lee, Jang-mi Choi, Nahye Kim, Seo-jin Park, Min-jae Park, Jeong-hyeon Lim, Young-Guk Na, Young G. Shin

**Affiliations:** College of Pharmacy and Institute of Drug Research and Development, Chungnam National University, 99, Daehak-ro, Yuseong-gu, Daejeon 34134, Korea; jinju.byeon.cnu@gmail.com (J.-J.B.); minho.park.cnu@gmail.com (M.-H.P.); seokho.shin.cnu@gmail.com (S.-H.S.); yuri.park.cnu@gmail.com (Y.P.); byungill.lee.cnu@gmail.com (B.i.L.); jangmi.choi.cnu@gmail.com (J.-m.C.); nahye.kim.cnu@gmail.com (N.K.); seojin.park.cnu@gmail.com (S.-j.P.); minjae.park.cnu@gmail.com (M.-j.P.); Jeonghyeon.Lim.cnu@gmail.com (J.-h.L.); youngguk@cnu.ac.kr (Y.-G.N.)

**Keywords:** ZM241385, Parkinson’s disease, A_2A_ receptor inhibitor, LC-qToF MS, pharmacokinetics, PBPK modeling

## Abstract

Parkinson’s disease is one of the most common neurodegenerative diseases. Adenosine regulates the response to other neurotransmitters in the brain regions related to motor function. In the several subtypes of adenosine receptors, especially, adenosine 2A receptors (A_2A_Rs) are involved in neurodegenerative conditions. ZM241385 is one of the selective non-xanthine A2AR antagonists with high affinity in the nanomolar range. This study describes the in vitro and in vivo pharmacokinetic properties of ZM241385 in rats. A liquid chromatography-quadrupole time-of-flight mass spectrometric (LC-qToF MS) method was developed for the determination of ZM241385 in rat plasma. In vivo IV administration studies showed that ZM241385 was rapidly eliminated in rats. However, the result of in vitro metabolic stability studies showed that ZM241385 had moderate clearance, suggesting that there is an extra clearance pathway in addition to hepatic clearance. In addition, in vivo PO administration studies demonstrated that ZM241385 had low exposure in rats. The results of semi-mass balance studies and the in silico PBPK modeling studies suggested that the low bioavailability of ZM241385 after oral administration in rats was due to the metabolism and by liver, kidney, and gut.

## 1. Introduction

Parkinson’s disease is one of the most common neurodegenerative diseases affecting more than 1% of the elderly population [1,2]. It is characterized by four symptoms such as rigidity, bradykinesia, tremor, and postural instability [3]. These symptoms are directly caused by dopamine deficiency which is due to the selective loss of dopaminergic (DA) neurons in the substantia nigra [4,5,6]. The treatment of Parkinson’s disease is focused on the use of dopamine replacement drugs such as levodopa (L-dopa) to compensate for the loss of dopamine. However, as the disease progresses and DA neurons are lost, the efficacy of L-dopa diminishes and motor fluctuation (such as wearing-off and on-off phenomena) and dyskinesia tend to appear in the PD patients [7,8,9]. In addition, it appears to be at increased risk for non-dopamine-related symptoms such as cognitive and psychiatric dysfunctions become more prominent, leading to long-term disability [10,11,12,13]. 

Adenosine is a neurotransmitter that regulates the response to dopamine and other neurotransmitters in the brain regions involved in motor function, mood, learning, and memory [14,15]. The adenosine-dopamine interactions have been studied in terms of their relevance to the treatment of central nervous system (CNS) disorders [16,17,18,19]. In the several subtypes of adenosine receptors, especially, adenosine 2A receptors (A_2A_Rs) are reported that they were upregulated in the brain under the stress condition and the blockade of these receptors showed brain neuro-protection effect in animal studies [20,21,22,23,24]. In other words, the blockade of A_2A_Rs would decrease the risk of brain disorders in different neurodegenerative conditions, such as ischemia, epilepsy, Parkinson’s, and Alzheimer’s disease. Recently, Nourianz (Istradefylline, Kyowa kirin) which is a selective A_2A_R antagonist, was approved as an add-on therapy to treat off period in Parkinson’s disease. Since A_2A_Rs are present in the striatum and the interaction between adenosine and dopamine receptors is intense, it has been presented as a non-dopaminergic strategy for the treatment of Parkinson’s disease [25,26,27]. The motor function was improved in different animal studies when the A_2A_Rs antagonists were administered alone or in combination with dopamine-mimetic drugs, levodopa, or dopamine agonists [21,22,23,24]. 

**Figure 1:**
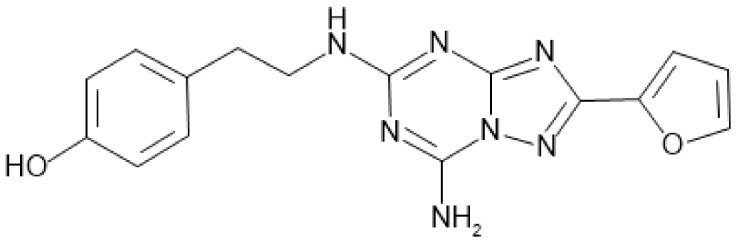
Structure of ZM241385.

ZM241385 is one of the potent non-xanthine adenosine A_2A_ receptor antagonists (Figure 1). It is a competitive A_2A_R antagonist with high affinity in the nanomolar range while it has low affinity at the other adenosine receptor subtypes (A_1_, A_2B_, and A_3_) [28]. Despite the favorable features of A_2A_R antagonist, ZM241385 only has been used as a reference drug in the development of A_2A_R antagonists in several studies. In addition, there has been no study about the pharmacokinetics (PK) for ZM241385 in animals as well as in human so far to our best knowledge. In this paper, in vitro and in vivo studies were conducted to characterize the PK properties of ZM241385 in rats and the parameter sensitivity analysis (PSA) using in silico physiologically based pharmacokinetic (PBPK) model was also performed to investigate the factors affect the exposure of ZM241385 after oral administration. 

## 2. Results and Discussions 

### 2.1. Method Qualification for the Determination of ZM241385 in Bioanalysis Samples.

The calibration curve was selected based on the analysis of the data by linear regression with weighted quadratic regression (1/x^2^). The mean correlation coefficient (r) value was >0.99 for the linearity of calibration curve ranged from 1.01 to 2222.22 ng/mL. Assay performance was determined by assessing the accuracy and precision of QC samples with three different concentrations (15.03, 165.29, and 1818.18 ng/mL). The accuracy and precision at three QC samples met the acceptance criteria of discovery research within ±25% except for the LLOQ (±30%) [29]. The intra-day precision at the three QC samples ranged from 0.45 to 12.64% and the intra-day accuracy ranged from 94.90 to 105.53% respectively. The inter-day precision at the three QC samples ranged from 2.44 to 8.28% and the inter-day accuracy ranged from 97.04 to 100.92% respectively (Table 1). The dilution integrity was evaluated by preparing dilution QC samples (10000 ng/mL) at five-fold dilutions. The result for dilution integrity is presented in Table 2.

All the results of stability studies are presented in Table 3. For short-term stability, the plasma samples were kept at room temperature for the time period expected routine sample preparation. The result showed that the ZM241385 in rat plasma was stable under the current experimental conditions. For freeze-thaw stability, the result indicated that the ZM241385 in rat plasma was stable for three freeze-thaw cycles when stored at −80 °C and thawed to room temperature. For long-term stability, ZM241385 in rat plasma was stable for 1 month at −80 °C and this result indicated that the samples of ZM241385 could be stored for 1 month. For post-preparative stability, the result showed that ZM241385 after the preparation procedure was stable for 24 h in auto-sampler condition and all LC-qToF MS analyzes were performed within 24 h. The results of stability studies represented that ZM241385 showed reliable stability behavior in rat plasma and auto-sampler within the acceptance criteria of ±25%. 

### 2.2. In Vivo Pharmacokinetic Study of ZM241385 in Rats

**Figure 2:**
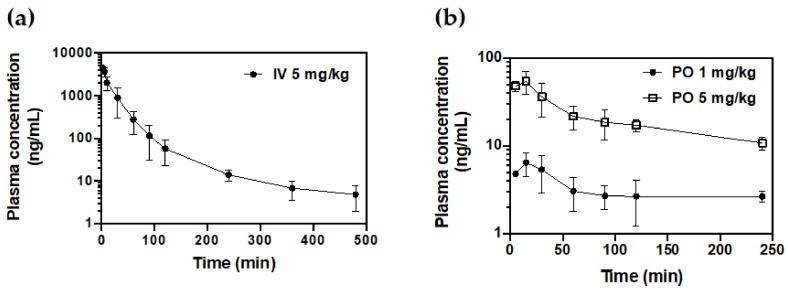
Mean concentration-time profiles of ZM241385 in rat after (**a**) intravenous (IV) administration at 5 mg/kg and (**b**) oral (PO) administration at 1 and 5 mg/kg.

The developed method was applied to determine the plasma concentration of ZM241385 in rat plasma after intravenous administration of 5 mg/kg and oral administration of 1 and 5 mg/kg. The PK profile of the mean plasma concentration versus time is shown in Figure 2 and the PK parameters calculated using a non-compartmental analysis method are presented in Table 4. 

The observed mean maximum plasma concentration (C_max_) after intravenous administration was 4458.03 ng/mL and the area under the curve (AUC_last_) from time 0 to last time point was 100,446.26 ng∙min/mL. The systemic clearance (Cl) and the volume of distribution at steady state (V_ss_) were estimated as 54.57 mL/min/kg and 1880.38 mL/kg, respectively. The observed mean maximum plasma concentration (C_max_) after oral administration was 6.67 and 58.29 ng/mL and the area under the curve from 0 min to the last measured time point was 1125.53 and 6599.69 ng∙min/mL at 1 and 5 mg/kg doses, respectively. These results showed that ZM241385 was rapidly eliminated in rats and the systemic clearance was almost similar to hepatic blood flow (55.2 mL/min/kg). The mean oral bioavailability of ZM241385 in rats was 6.09% and this value indicated that ZM241385 had low bioavailability.

### 2.3. In Vitro Metabolic Stability in Liver Microsome and S9 Fraction

The metabolic stability of ZM241385 was assessed with liver microsome and S9 fractions to evaluate the main cause of high clearance of the drug and it was based on the extent of ZM241385 reduction due to hepatic metabolism over time. The results of in vitro metabolic stability in the NADPH-fortified liver microsomes as well as the NADPH- and UDPGA-fortified S9 fractions were shown in Table 5.

The extrapolated in vivo hepatic clearances of ZM241385 in rat liver microsomes with NADPH was 25.93 mL/min/kg and in S9 fractions with NADPH and UDPGA was 27.16 mL/min/kg. These results imply that ZM241385 might primarily undergo microsomal metabolism rather than cytosolic metabolism in rat. Also, since this calculated in vivo hepatic clearance was almost half of hepatic blood flow in both microsomes and S9 fractions, ZM241385 would be moderately cleared by hepatic metabolism in rat in vivo study. However, in previous in vivo PK study, the systemic clearance of ZM241385 was nearly similar to the hepatic blood flow. Taken together, these results show that the observed in vivo clearance is greater than the extrapolated clearance from the in vitro metabolic stability study, which suggests that ZM241385 has extra elimination pathways such as clearance in kidney or gut in addition to the hepatic clearance. 

### 2.4. Semi-Mass Balance Study of ZM241385 in Bile-Duct Cannulated Rat

The semi-mass balance study using bile-duct cannulated rat was performed to evaluate the cause of low exposure of ZM241385 after oral administration. Bile duct cannulation (BDC) studies are usually conducted to determine the absorption, distribution, metabolism, and excretion profiling of drugs. The recoveries as a percentage of the administered dose over time following oral ZM241385 are summarized in Table 6.

After an oral dose of 5 mg/kg ZM241385 in BDC rat, 18.76% of the dose was recovered for 48 h. The cumulative recoveries of total ZM241385 in urine, bile, and feces were 2.56%, 5.68%, and 10.06%, respectively. The oral bioavailability depends on many factors, such as dissolution, drug stability in the gastrointestinal tract, absorption, the rate of passage through the gut wall, and the pre-systemic metabolism in the gut wall and liver [30]. This result indicated that the percentage of parent ZM241385 excreted without absorption was 10.06%. Therefore, the remaining 89.94% of the drug was absorbed and then excreted to 2.56% as urine and 5.68% as bile after reaching the systemic circulation through metabolism. In other words, the low oral exposure of ZM241385 is mainly caused by the metabolism in liver and other organs rather than the absorption issue in GI tract or excretion through bile and urine.

### 2.5. PBPK Modeling to Predict the Concentration in Brain and Evaluate the Poor Nioavailability of ZM241385

**Figure 3:**
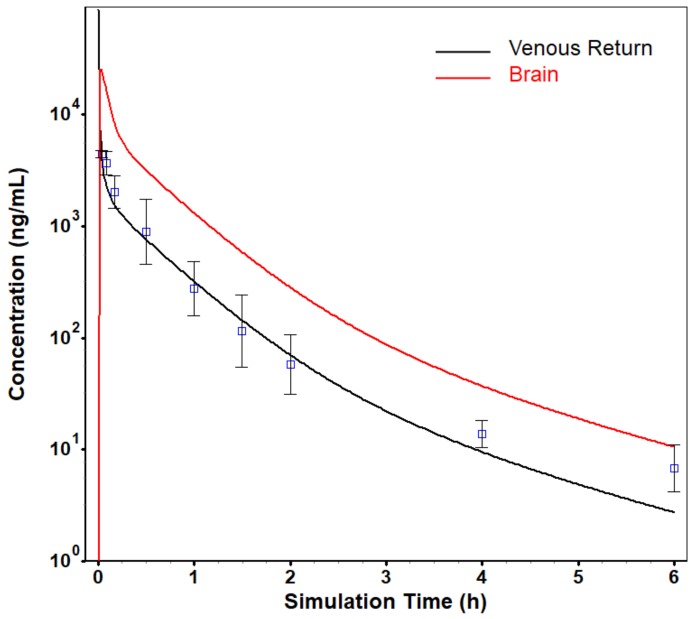
The simulated and observed plasma concentration-time profile and the simulated brain concentration-time profile of ZM241385 in rat after intravenous (IV) administration at 5 mg/kg using GastroPlus™ (red; brain, black; plasma).

The in silico PBPK model-based approach using GastroPlus™ was applied to determine which factors were involved in low exposure of ZM241385 after oral administration. The PBPK model is a mechanism-based approach that integrates three sources; system-specific properties, drug properties, and anatomical arrangement of tissues or organs [31]. GastroPlus™, which is a PBPK software tool, is generally used to predict and simulate the PK profile of the drugs in preclinical and clinical setting. Since the absorption, distribution, metabolism, and elimination processes are complex and involve a large number of factors, an integrated approach is required. Therefore, the PSA using PBPK model was performed to assess the influence of these factors. The PBPK model was established based on the in vivo PK parameters and the predicted in vivo hepatic clearance. The predicted and observed PK profiles after IV administration of ZM241385 were presented in Figure 3 and the PK parameters were presented in Table 7. Since ZM241385 is a drug candidate for the treatment of Parkinson’s disease, the simulation of ZM241385 in the brain was performed based on this PBPK model and the predicted PK profile in the brain was also presented in Figure 3. 

The PBPK model was assessed by calculating the fold error between the observed and the predicted data. The predicted AUC was within the 0.5–2.0 range (two-fold error) [32,33]. Based on the simulation results in the brain, ZM241385 would be distributed to the brain and the brain-to-plasma ratio would exceed 1.

**Figure 4:**
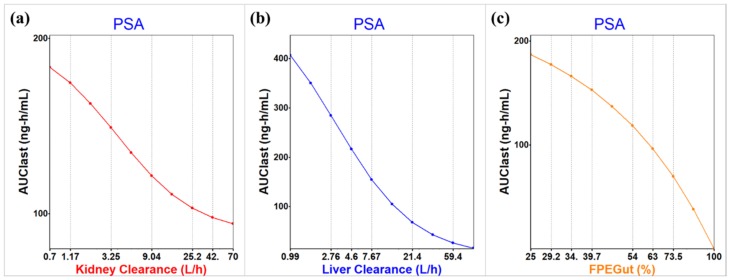
The parameter sensitivity analysis (PSA) for three factors influencing low bioavailability in the oral administration of ZM241385; (**a**) kidney clearance, (**b**) liver clearance, and (**c**) first pass effect in gut.

Based on this PBPK model, PSA was performed using GastroPlus™ to evaluate the factors influencing low bioavailability after the oral administration of ZM241385. First of all, various factors related to the pharmacokinetic parameters representing drug exposure (AUC_last_) were assessed. As a result, three factors were considered to mainly contribute to the low exposure of ZM241385 after the oral administration; kidney clearance, liver clearance, and the intestinal first-pass effect (FPE). The PSA results were shown in Figure 4. Based on these results, the metabolism in kidney, liver, and gut would have a significant impact on the low bioavailability of ZM241385.

## 3. Materials and Methods

### 3.1. Chemicals and Reagents

ZM241385 was purchased from MedChem Express (Monmouth Junction, NJ, USA). Verapamil (internal standard; ISTD) was purchased from Sigma-Aldrich (St Louis, MO, USA). Nicotinamide adenine dinucleotide phosphate reduced (NADPH) regenerating system solutions (A and B) and uridine 5′-diphosphoglucuronic acid triammonium salt (UDPGA) were purchased from Corning Incorporated (Corning, NY, USA) and Sigma-Aldrich (St Louis, MO, USA), respectively. The liver microsome was purchased from Corning Incorporated (Corning, NY, USA). The S9 fraction was purchased from Thermo Fisher Scientific Inc. (Waltham, MA, USA). All other chemicals were commercial products of analytical or reagent grade and used without further purification. 

### 3.2. In Vivo Pharmacokinetics Study in Rats

All animal studies were performed in accordance with the “Guidelines in Use of Animal” established by the Chungnam National University Institutional Animal Care and Use Committee (Daejeon, Korea). This study was approved by the Chungnam National University Institutional Animal Care and Use Committee (No. CNU-01104). The study was performed in male Sprague-Dawley (SD) rats (Samtako Biokorea, Gyeong-gi, Korea) weighting 280–300 g. The rats were randomly divided into three groups of three rats. ZM241385 was administered intravenously in the femoral vein or orally at a dose of 5 mg/kg. All rats were fasted for 24 h prior to drug administration. Blood samples were collected at 2, 5, 10, 30, 60, 90, 120, 240, 360, and 480 min following intravenous administration or 5, 15, 30, 60, 90, 120, and 240 min following oral administration. Whole blood samples were centrifuged at 12,000 rpm for 5 min and the supernatant was transferred to another tube and then stored at −20 °C until further analysis. After plasma sample preparation, the concentrations of ZM241385 in plasma were determined using an LC-qToF MS/MS method. 

The pharmacokinetic parameters were calculated based on non-compartmental analysis (NCA) using the Phoenix WinNonlin™ software (version 8.1.0; Pharsight Corporation, Mountain View, CA, USA). At least four points of plasma concentrations were used to calculate terminal half-life.

### 3.3. In Vitro Metabolic Stability in Rat Liver Microsome and S9 Fraction

The in vitro metabolism of ZM241385 was investigated in rat liver microsome and S9 fraction. The drug was incubated for 0, 15, and 30 min at 37 °C in rat NADPH-fortified liver microsomes or both NADPH- and UDPGA-fortified liver S9 fractions and the reaction was stopped by adding 100% ACN containing 100 ng/mL ISTD. 

The drug remaining percent was used for the determination of the in vitro T_1/2_. The slope (k) of log percentage remaining versus incubation time was used in the conversion to in vitro T_1/2_ values by in vitro T_1/2_ = −0.693/k. Extrapolation to Cl_int_ was done using the following formula in microsome and S9 fraction, respectively [34,35].

(1) $$Cl_{int,microsome}\;=\;\left(\frac{0.693}{t_{1/2}}\;\right)\times \left(\frac{microsome\;volume\;\left(mL\right)}{amount\;microsomal\;protein\;in\;incubation\left(mg\right)}\right)\times \left(\frac{44.8\;mg\;microsomal\;protein}{g\;liver}\right)\times \left(\frac{40\;g\;liver}{kg\;body\;weight}\right)$$

(2) $$Cl_{int,\;S9}\;=\;\left(\frac{0.693}{t_{1/2}}\;\right)\times \left(\frac{S9\;volume\;\left(mL\right)}{amount\;S9\;fraction\;protein\;in\;incubation\left(mg\right)}\right)\times \left(\frac{121\;mg\;S9\;fraction\;protein}{g\;liver}\right)\times \left(\frac{40\;g\;liver}{kg\;body\;weight}\right)$$

Predicted in vivo hepatic clearance (Cl_H_) was calculated using well-stirred model [36]. In the “well stirred” model, it is assumed that the liver is a single well-stirred compartment and the distribution equilibrium is achieved so rapidly that the drug in blood is in equilibrium with the unbound drug within the liver. The in vivo hepatic clearance (Cl_H_) extrapolated from intrinsic clearance (Cl_int_) using “well stirred” model is expressed as shown in formula below.

(3) $$Cl_{H}\;=\;\left(\frac{Q_{h}\times Cl_{int}}{Q_{h}+Cl_{int}}\right)$$

where the Q_h_ is the liver blood flow of 55.2 mL/min/kg for rats [34]. 

### 3.4. Semi-Mass Balance Study in Rats

The purpose of the semi-mass balance study in rat was to investigate the root cause of low exposure for ZM241385 following an oral administration at 5 mg/kg for three rats. Urine, bile, and feces samples were collected for 48 h from rat metabolic cages (JeungDo Bio & Plant co., Seoul, Korea) designed for separate collection of urine and feces. The collected urine samples were diluted 1:1 in 30% ACN. The collected feces samples were diluted in four volumes of PBS (five-fold dilution) and then homogenized. The obtained samples were transferred to Eppendorf tube and then treated following the study sample preparation procedures.

### 3.5. PBPK Modeling in Rats Using GastroPlus™

The physiologically based pharmacokinetic (PBPK) model by GastroPlus™ (version 9.6; Simulations Plus, Inc., Lancaster, CA, USA) was established based on the in vivo PK profile and in vivo systemic clearance. Liver and kidney were assumed to be main elimination organs in the PBPK models. If the predicted in vivo hepatic clearance extrapolated from in vitro metabolic stability result was lower than the observed in vivo systemic clearance, extra hepatic clearance such as renal clearance was considered. After the PBPK model was established, simulation in the brain was performed to predict the exposure of ZM241385 in the target organ. Parameter sensitivity analysis (PSA) was performed using PSA module in GastroPlus™ to search for the main factors influencing low bioavailability in the oral administration of ZM241385.

### 3.6. Preparation of Calibration Standard (STD) and Quality Control (QC) Samples

The stock solution of ZM241385 was prepared by dissolving in DMSO at a concentration of 1 mg/mL. Then the 0.1 mg/mL sub-stock solution was serially diluted with DMSO to prepare the calibration standard working solutions. An aliquot of 4 µL of each calibration standard (STD) and quality control (QC) solution was spiked to 20 µL of rat blank plasma. Then all the STD and QC samples were treated following the study sample preparation procedures. The calibration curves were constructed y weighted quadratic regression (1/x^2^) with a correlation coefficient (r) value ≥0.99 ranged from 1.01 to 2222.22 ng/mL. Assay performance was assessed by accuracy and precision of QC samples with three different concentrations (15.03, 165.29, and 1818.18 ng/mL). 

### 3.7. Preparation of Study Samples

An aliquot of 4 µL of make-up solution (DMSO) was spiked to 20 µL of rat plasma study sample. Then, 100 µL of ACN containing 100 ng/mL ISTD was added to the mixture for protein precipitation. After vortexing for 1 min, the mixture was centrifuged at 12,000 rpm for 5 min. The supernatant was transferred to Eppendorf tube and diluted three times with distilled water. The diluted sample was transferred to LC vial for LC-qToF MS/MS analysis. 

### 3.8. LC-MS Condition

The liquid chromatography-mass spectrometry (LC-MS) system consisted of two Shimadzu LC-20AD pumps, a Shimadzu CBM-20A HPLC pump controller (Shimadzu Corporation, Columbia, MD, USA), a CTC HTS PAL auto-sampler (LEAP Technologies, Carrboro, NC, USA) and a Triple TOF™ 5600 mass spectrometer system (Sciex, Foster City, CA, USA). Triple TOF™ 5600 mass spectrometer with an electrospray ionization source (ESI) was used to perform the high resolution experiment. ZM241385 and ISTD were separated on a Phenomenex® Kinetex XB-C18 column (2.6 µm, 2.1 × 50 mm) maintained at 55 °C. The mobile phase was composed of (A) 0.1% formic acid in water and (B) 0.1% formic acid in acetonitrile delivered at a flow rate of 0.4 mL/min. The LC gradient for ZM241385 analysis is summarized in Table 8. 

The Triple TOF™ 5600 mass spectrometer equipped with an ESI was operated in the positive ion mode. High-purity nitrogen gas was used for the nebulizer/Duospray™ and curtain gases. The instrument conditions were optimized as follows. Source temperature was set at 500 °C with a curtain gas flow of 30 L/min (GS1 and GS2 both 50). The compound specific voltages such as declustering potential (DP) and collision energy (CE) were optimized as 160 V and 37 V for ZM241385; 125 V and 30 V for ISTD. The mass transitions of precursor-to-product ions monitored were *m/z* 338.1→121.1 for ZM241385 and *m/z* 455.2→165.2 for ISTD, respectively. Data processing was performed using the Analyst® TF 1.6 and MultiQuant™ 3.0.3 software (Sciex, Foster City, CA, USA) 

## 4. Conclusions

ZM241385 is one of the selective A_2A_R antagonist and it has been used as a reference drug in many studies for A_2A_Rs. Inhibition of A_2A_Rs shows neuro-protection effect and it is proposed as an alternative non-dopaminergic strategy for the treatment of Parkinson’s disease. Although it is potent for A_2A_R, there has been no study so far about the in vitro and in vivo PK for ZM241385 to the best of our knowledge. The purpose of this paper is to characterize the ADME/PK properties of ZM241385 in rats based on the in vitro, in vivo, and in silico studies for the first time. 

First of all, a selective and reproducible LC-qToF MS/MS method of ZM241385 was developed to determine the drug concentrations in rat plasma. The range of calibration curve was 1.01 to 2222.22 ng/mL with a correlation coefficient ≥0.99. The drug in rat plasma was stable under the short-term, long-term, and freeze-thaw condition and achieved the dilution integrity. This method was successfully applied to analyze the in vitro metabolic stability samples, and the in vivo PK samples and the semi-mass balance study samples after intravenous and oral administration of ZM241385 in rat. 

The in vivo PK studies for ZM241385 in rats showed that ZM241385 has high clearance and poor bioavailability. The in vitro metabolic stability studies in microsomes as well as S9 fractions were performed to determine the contribution of hepatic metabolism to systemic clearance and the hepatic clearance values extrapolated from these studies were comparable (25.93 and 27.16 mL/min/kg, respectively). This result indicated that ZM241385 is moderately cleared by hepatic metabolism and probably another clearance pathway is involved in the in vivo systemic clearance of the drug. Also, in vivo semi-mass balance study and parameter sensitivity analysis using the PBPK modeling were conducted to evaluate the contribution to the poor exposure of ZM241385 in rat. The semi-mass balance results presented that approximately 8.24% of ZM241385 was excreted as a parent form after oral administration for 48 h. In addition, approximately 10.06% of ZM241385 was excreted as a parent form without absorption for 48 h. These results indicated that only a small amount of unchanged drug reached the systemic circulation and then was excreted through urine or bile. The PBPK modeling and simulation were performed to find the factors affecting the low exposure. The factors affecting the exposure of ZM241385, especially AUC_last_, were investigated using the parameter sensitivity analysis of GastroPlus™. As a result, kidney clearance, liver clearance, and intestinal first-pass effect (FPE%) appear to play a significant role in the low exposure of ZM241385, which implies that the main root causes of low bioavailability of ZM241385 were possibly due to liver, kidney, and gut metabolism. 

In conclusion, the in vitro, in vivo, and in silico pharmacokinetic properties of ZM241385 were evaluated for the first time in this study. The results of these studies indicate that ZM241385 has high clearance and low bioavailability in rats because of the metabolism in liver, kidney, and gut. This study would be able to support to identify the characteristics of PK as a tool compound when developing ZM241385 and its related compounds in Parkinson’s disease research areas. Further studies such as metabolites identification analysis for ZM241385 would be warranted to lead to more drug-like structures for ZM241385 with improved ADME/PK property. 

## Figures and Tables

**Table 1:** Intra/inter-day accuracy and precision of ZM241385 in quality control samples at three different levels (*n* = 3 for intra-day accuracy and precision; *n* = 9 for inter-day accuracy and precision).

Run no.	Statistics	QC Low (15.03 ng/mL)	QC Medium (165.29 ng/mL)	QC High (1818.18 ng/mL)
1	Mean concentration	14.86	159.01	1845.76
	Accuracy (%)	98.85	96.20	101.52
	Precision (%, CV)	6.55	0.45	5.32
2	Mean concentration	14.70	156.85	1814.97
	Accuracy (%)	97.83	94.90	99.82
	Precision (%, CV)	2.45	0.59	4.09
3	Mean concentration	15.86	165.31	1844.24
	Accuracy (%)	105.53	100.01	101.43
	Precision (%, CV)	12.64	0.77	5.79
Inter-day	Mean concentration	15.14	160.39	1834.99
	Accuracy (%)	100.74	97.04	100.92
	Precision (%, CV)	8.28	2.44	4.96

Quality control (QC); coefficient of variation (CV).

**Table 2:** The dilution integrity assessment of ZM241385 in rat plasma (*n* = 3).

Assessment	Dilution Factor	Statistics	Dilution QC (10,000 ng/mL)
Dilution integrity	5-fold	Mean concentration	9741.65
		Accuracy (%)	97.42
		Precision (%, CV)	2.39

**Table 3:** The stability assessments of ZM241385 in rat plasma (*n* = 3).

Assessment	Statistics	QC Low (15.03 ng/mL)	QC Medium (165.29 ng/mL)	QC High (1818.18 ng/mL)
Short-term stability	Mean concentration	16.77	169.34	1601.21
	Accuracy (%)	111.55	102.45	88.07
	Precision (%, CV)	11.15	9.72	5.39
Long-term stability	Mean concentration	16.90	182.65	1905.82
	Accuracy (%)	112.47	110.50	104.82
	Precision (%, CV)	5.35	4.21	1.93
Freeze-thaw stability	Mean concentration	15.16	164.66	1813.88
	Accuracy (%)	100.90	99.62	99.76
	Precision (%, CV)	2.24	3.13	1.76
Post-preparative stability	Mean concentration	16.43	189.27	2091.45
	Accuracy (%)	109.28	114.51	115.03
	Precision (%, CV)	2.49	2.53	0.23

**Table 4:** Pharmacokinetic parameters of ZM241385 after IV administration at 5 mg/kg and PO administration at 1 and 5 mg/kg in rat.

	Dose (mg/kg)	t_1/2_ (min)	C_max_ (ng/mL)	AUC_last_ (ng∙min/mL)	Cl (mL/min/kg)	V_ss_ (mL/kg)	BA (%)
IV	5	89.42 ± 18.14	4458.03 ± 282.51	100,446.26 ± 41,828.36	54.57 ± 18.34	1880.38 ± 618.10	-
PO	1	175.09 ± 60.98	6.67 ± 1.73	1125.53 ± 239.37	-	-	5.60 ± 0.96
	5	144.48 ± 20.87	58.29 ± 12.64	6599.69 ± 1232.90	-	-	6.57 ± 0.26

Half-life (t_1/2_); maximum concentration (C_max_); area under the plasma concentration vs. time curve from 0 to last time point (AUC_last_); systemic clearance (Cl); volume of distribution at steady state (V_ss_); bioavailability (BA).

**Table 5:** In vitro microsomal metabolic stability result for ZM241385 in rat liver microsome and S9 fraction (*n* = 3).

Matrix	t_1/2_ (min)	Cl_int_ (mL/min/kg)	Cl_H_ (mL/min/kg)
Liver microsome	50.80 ± 6.82	48.89 ± 2.64	25.93 ± 1.08
Liver S9 fraction	125.44 ± 1.86	53.48 ± 0.81	27.16 ± 0.21

Intrinsic clearance (Cl_int_); extrapolated in vivo hepatic clearance (Cl_H_).

**Table 6:** Percentage of administered dose of ZM241385 excreted in urine, bile and feces following oral administration of 5 mg/kg in bile duct-cannulated rat.

Matrix	Time (h)	Drug Amount (μg)	Excretion (%)
Urine	0–48	35.85 ± 29.35	2.56 ± 2.10
Bile	0–48	79.49 ± 22.03	5.68 ± 1.57
Feces	0–48	140.83 ± 1.71	10.06 ± 0.12

**Table 7:** The simulated and observed C_max_ and AUC_last_.

PK Parameters	Observed	Predicted
C_max_ (ng/mL)	4458.0 ± 282.5	4410.0
AUC_last_ (ng∙hr/mL)	1674.1 ± 697.1	1672.2

**Table 8:** Gradient elution timetable for LC separation.

Time (min)	Mobile Phase B (%)
0	10
0.5	10
1.1	95
1.5	95
1.6	10
3.0	10
